# Selenium-induced structural reorganization of polysaccharides from blackened jujube pomace enhances immunomodulatory activity

**DOI:** 10.3389/fnut.2026.1791870

**Published:** 2026-03-17

**Authors:** Min Zhao, Xin Sun, Yuxiao Wang, Lin Gao, Rentang Zhang

**Affiliations:** 1 College of Food Science and Engineering, Shandong Agricultural University, Tai'an, Shandong, China; 2 Laoling Tailetang Food Technology Co., Ltd., Dezhou, China; 3 Laoling Healthy Food Industry Technology Research Institute, Dezhou, China

**Keywords:** characterization, immunomodulatory activity, polysaccharides, process optimization, selenated polysaccharides

## Abstract

**Introduction:**

Natural polysaccharides exhibit promising pharmacological potential in functional foods. However, their structural heterogeneity and limited bioactivity hinder further applications.

**Methods:**

In this study, a polysaccharide from blackened jujube pomace was selenylated via the conventional HNO_3_-Na_2_SeO_3_ route, with systematic optimization of reaction parameters to improve controllability, yielding selenium-enriched polysaccharides (BJPP-Se).

**Results and Discussion:**

Multimodal characterization confirmed successful selenylation, with a reduction in molecular weight from 88.26 kDa to 74.32 kDa and an increase in crystallinity. Density functional theory calculations identified two distinct Se (IV) coordination modes, with one involving Se (IV) as a bridging atom linking two monosaccharide units and the other restricting Se (IV) coordination to a single monosaccharide unit. BJPP-Se has much stronger immunomodulatory performance than native polysaccharides in cyclophosphamide suppressed immunosuppressed mice. Selenium-induced structural reorganization is a very good example of how to design bioactive polysaccharides. This work supports the potential for valorizing polysaccharides from black jujube pomace; however, large-scale techno-economic feasibility and cost–benefit assessment are beyond the scope of this study and warrant future work with clearly defined boundaries and engineering-scale datasets.

## 1 Introduction

Immunodeficiency: Not having a strong immune system and being weak, because you cannot make enough antibodies and/or immune cells (1). Beyond mere infection susceptibility, contemporary understanding frames many primary immunodeficiencies as diseases of immune dysregulation, often presenting with autoimmunity, hyperinflammation, and malignancy risk (2) The administration forms at the present time include immunoglobulin replacement, avoiding sicknesses and giving assistance (2). But these methods usually need to be used for a long time or even life and maybe cannot make the immune system be in a peaceful situation completely (3). In this context, bioactive food-derived components, particularly polysaccharides, have garnered substantial interest as potential immunomodulatory agents for functional food applications.

Indeed, polysaccharides from natural sources are recognized for their ability to interact with the immune system. Pattern recognition receptors on macrophages and DCs can also be recognized, changing cell activation and cytokine secretion (4). Key pathways are usually TLR-linked signaling, NF-κB, and MAPK modules (5). Polysaccharides might also have an impact on antigen presentation and downstream T cell reactions. In other settings, they may form trained-immunity like features (4). However, their practical application is frequently hampered by inherent limitations, including structural heterogeneity, which complicates quality control and reproducibility, and often modest *in vivo* efficacy, especially within the complex, oxidative stress-prone microenvironment of immunodeficiency (4).

To overcome these barriers, structural modification strategies, such as selenylation, have emerged as a powerful tool to enhance and tailor polysaccharide bioactivity. Selenium modifications are practical strategies. Selenium, as an important nutrient for supporting immunity, redox imbalance and selenoproteins (6). The strategic integration of selenium into polysaccharide scaffolds creates novel conjugates that synergistically combine the structural benefits of the polysaccharide with the potent biological activity of selenium. Selenium-polysaccharides, which are made by combining a polysaccharide structure with selenium-related biological activity. And they also indicate it can also be seen more frequently that selenium polysaccharide possesses far stronger antioxidant and immunoregulatory functions than does either parent polysaccharide on its own or selenium on its own (7). Therefore, the development of well-characterized selenium-polysaccharides from sustainable sources represents a rational approach to creating advanced nutraceutical ingredients.

Agro-industrial byproducts offer an eco-friendly and valuable resource for such biopolymer extraction. Blackened jujube pomace, a significant residue from jujube processing, is rich in polysaccharides but remains underutilized. Exploiting this byproduct aligns with circular economy principles and adds value to the food production chain. Meanwhile, the nitric acid–sodium selenite (HNO_3_-Na_2_SeO_3_) route has been widely used for polysaccharide selenylation; however, many studies provide limited process control, which may contribute to product heterogeneity. Therefore, improving controllability through systematic optimization of key reaction parameters is important for obtaining more reproducible selenium-enriched polysaccharides and for linking structure to function.

Accordingly, this study aimed to prepare and characterize a selenium-enriched polysaccharide (BJPP-Se) from blackened jujube pomace using the conventional HNO_3_-Na_2_SeO_3_ selenylation route under systematically optimized conditions. We combined sequential purification (ion-exchange and gel chromatography) with single-factor optimization of reaction time, temperature, and reactant ratio, followed by multi-technique characterization (SEM-EDS, FT-IR, XPS, XRD, TG, CD) to probe selenium-associated structural changes. Along with investigation of immunomodulatory activity, we posit that the selenium-induced structural reorganization, particularly at the molecular coordination level, is the fundamental driver for the significantly enhanced immune-regulatory activity. This study elucidates the selenium-induced molecular reorganization mechanisms and establishes a robust structure-activity relationship, providing a mechanistic framework to guide the rational design of advanced nutraceuticals and bridge the knowledge gap between polysaccharide engineering and the innovation of functional foods and pharmaceuticals. A systematic comparison of this work with representative recent studies regarding Se incorporation, characterization, and biological activities is provided in Table 1.

**Table 1:** Systematic comparison of BJPP-Se with representative recent literature regarding Se incorporation, characterization, and biological activities.

Study	Se incorporation method	Characterization methods	Biological activities
BJPP-Se	HNO_3_-Na_2_SeO_3_	SEM-EDS, UV–Vis, FT-IR, XRD, TG/DTG, CD, Congo red, Mw & monosaccharide profiling; XPS; DFT	CTX-immunosuppressed mice: organ index, histology, CBC, cytokines (IL-2/IL-6/IFN-γ/TNF-α), IgE/IgG, T-cell subsets (CD3/CD4/CD8), apoptosis.
Se-GEP (60)	HNO_3_-Na_2_SeO_3_	UV, FT-IR, NMR, SEM–EDX, Mw, particle size and zeta potential analysis, I_2_-KI, Congo red	Immunomodulatory effects reported on RAW264.7 macrophages and CTX-treated mice.
Se-PFPS (61)	HNO_3_-Na_2_SeO_3_	FT-IR, UV, SEM, particle size, Mw, XRD, TGA	Primarily physicochemical/structural comparison.
ASPS-e1-Se (62)	HNO_3_-Na_2_SeO_3_	UV, FT-IR, Mw & monosaccharide profiling	Enhanced anti-tumor immunity through gut-microbiota modulation and cytokine regulation.

## 2 Materials and methods

### 2.1 Materials and chemicals

Blackened jujube pomace was purchased from Laoling Tai Le Tang Food Technology Co., Ltd. (Dezhou, Shandong, China). Sodium selenite and 3,3’-Diaminobenzidine (DAB) were purchased from Shanghai Aladdin Biochemical Technology Co., Ltd. (Shanghai, China). Cyclophosphamide and levamisole hydrochloride were obtained from commercial biochemical suppliers in Shanghai, China.

### 2.2 Preparation of polysaccharide selenium

The crude polysaccharide in blackened jujube pomace was extracted by hot water leaching, and after alcohol precipitation, the fat-soluble substances were removed by petroleum ether, and the proteins were removed by the Sevage method. The crude extract was then further purified using DEAE-Bestarose FF anion-exchange and gel-filtration chromatography (Chromdex 75PG). And in this experiment, fraction II was used for further studies and subsequently referred to collectively as BJPP (Supplementary Figures 1, 2).

The selenium polysaccharide was synthesized through a modified nitric acid–sodium selenite method. In summary, 1 g of purified polysaccharide was dissolved in 100 mL of HNO_3_ solution (0.095 mol/L, equivalent to 0.6% w/v) and stirred at room temperature for 30 min. Then Na_2_SeO_3_ (5.78 mmol, 1.00 g) and BaCl_2_ solution (1.0 mol/L, 100 mL) were added. The mixture was incubated at 75 °C for 8 h. After completion, the reaction mixture was neutralized with NaOH (1.0 mol/L). To remove residual Ba^2+^, Na_2_SO_4_ solution (1.0 mol/L, 100 mL) was added and the precipitate was removed by centrifugation (8). The supernatant was transferred into a dialysis bag (MWCO: 500 Da) and dialyzed against distilled water at 4 °C for 96 h with water changes every 6 h to remove small-molecule salts and residual reagents. The dialysate was then concentrated and lyophilized to obtain BJPP-Se (9). Selenium was determined using the 3,3′-diaminobenzidine colorimetric method (10). This product was designated BJPP-Se.

### 2.3 Investigation of BJPP-Se under different factors

Single-factor experiments were carried out to investigate the preparation of polysaccharide–selenium complexes. Fixed reaction conditions and changed the reaction time from 2.0 to 10.0 h, and the reaction temperature from 50 to 90 °C. At this time, the mass ratio of polysaccharide to Na_2_SeO_3_ was 2:8, 4:6, 5:5, 6:4, 8:2. The complexes were made in the same way, and then the amount of selenium in each sample was found.

### 2.4 Characterization analysis

Scanning electron microscope (ZEISS Sigma360, Germany) is used to observe the surface morphology of BJPP and BJPP-Se. The surface elemental composition of the BJPP and BJPP-Se is analyzed with energy dispersive spectroscopy (EDS) (9) in SEM.

UV–visible spectra were recorded with spectrophotometer (PERSEE T700AS, Beijing) in the range of 190–900 nm at room temperature (11). Crystalline state of the polysaccharides was measured with an X-ray diffractometer (Ultima IV, Kuraray CO., Ltd., Tokyo, Japan). Parameters: Cu target, Kα radiation, 40 kV, 40 mA, 2θ 10°-80°, scanning speed of 3° min^−1^ (12). The FT-IR spectra of the samples were analyzed by a Fourier transform infrared spectrophotometer (Thermo NICOLETiS10, USA) (13), and determined in a wavenumber range of 4,000–400 cm^−1^.

### 2.5 TG analysis

TG analysis of BJPP-Se and BJPP was performed by using a thermogravimetric analyzer (TA TGA 550, USA), with a program temperature of 700 °C and a heating rate of 10 °C/min. All determinations were conducted under a nitrogen atmosphere (8).

### 2.6 CD analysis and Congo red experiment

BJPP and BJPP-Se solutions were analyzed using circular dichroism spectroscopy (JASCO J-1500, Japan) for CD analysis in the scanning wavelength range of 190–330 nm (14).

1 mL of BJPP and BJPP-Se solutions (1.0 mg/mL) were transferred to a test tube. Then, 1 mL of Congo red solution (80 μmol/L) along with varying volumes of 1 mol/L NaOH solution and distilled water were added, adjusting the final NaOH concentrations to 0–0.5 mol/L (15). The UV–Vis absorption spectra were then recorded in the wavelength range of 300–700 nm.

### 2.7 Molecular weight determination and monosaccharide composition analysis

The samples were dissolved in 0.1 M NaNO_3_ aqueous solution (containing 0.02% NaN_3_, w/w) at a final concentration of 1 mg/mL and filtered through a filter with a pore size of 0.45 μm for online detection (13). The column temperature was 45 °C, the injection volume was 100 μL, and the mobile phase A (0.02% NaN_3_, 0.1 M NaNO_3_) was used at a flow rate of 0.6 mL/min with isocratic elution for 75 min.

The Thermo ICS 5000+ ion chromatography system (ICS 5000+, Thermo Fisher Scientific, USA) was utilized to analyze and detect monosaccharide fractions, featuring an electrochemical detector. 1 mL of 2 M TFA acid solution was added and heated at 121 °C for 2 h. Add sterile water to dissolve the sample, then transfer it to a chromatographic vial for measurement (15).

### 2.8 X-ray photoelectron spectroscopy (XPS) analysis

The surface elemental composition and oxidation states of BJPP and BJPP-Se were examined by XPS (Thermo Scientific K-Alpha, USA). Full-spectrum and narrow-band analyses were performed at passband energies of 160 eV and 40 eV, respectively. The binding energy scale was calibrated using C 1 s (284.6 eV) as the reference (16).

### 2.9 The coordination pattern of se(IV) with polysaccharide

Density functional theory (DFT) calculations were performed with Gaussian 16 C01 (17). B3LYP (18) functional was used. Grimme’s dispersion correction (19) with Becke-Johnson damping (20) was applied. 6-31G* basis set was used on all atoms. IEFPCM implicit solvent model (21) was used (water as continuum).

### 2.10 Immunomodulatory activity of BJPP and BJPP-Se

#### 2.10.1 Animal preparation and experimental design

Female ICR mice, approximately six to 8 weeks old and weighing around 20 g, were obtained from Shandong Pengyue Co., Ltd. All animal experiments strictly adhered to the guidelines for humane use and care of animals established by the Animal Ethics Committee of Shandong Agricultural University (Approval No. SDAUA-2025-036). Mice were housed in a specific pathogen-free (SPF) environment under a 12-h light–dark cycle, with temperature maintained at 22–24 °C and humidity at 50–60%. They had free access to water and food. After a 7-day acclimation period, mice were randomly divided into 6 groups, each comprising 12 mice. One group of healthy mice served as the normal control (NC) group, receiving daily saline injections for 10 consecutive days. From Day 1 to Day 3, the other five groups received intraperitoneal injections of cyclophosphamide (CTX) at 80 mg/kg body weight/day. From Day 4 to Day 10, mice received treatment at the doses shown in Supplementary Figure 3. CTX (0.2 mL) was administered via intraperitoneal injection. Other compounds were administered via gavage in a 0.2 mL solution. Twenty-four hours after the final dose, animals were weighed and then euthanized by decapitation.

#### 2.10.2 Changes in body weight and immune organ index

Mice were weighed periodically during the experiment. After euthanasia, the thymus and spleen were carefully dissected out. Each organ was rinsed with saline, blotted dry with absorbent paper, and weighed immediately. Organ indices were then calculated using the formula below (22):


$$\text{Immune organ index}\;(\mathrm{mg}/g)=\frac{\text{weight of immune organs}\;}{\text{body weight}\;}$$

#### 2.10.3 Histological examination of mouse spleen and thymus

Tissue sections of the mice’s spleen and thymus were first fixed in 4% neutral formaldehyde. The samples were stained with hematoxylin and eosin. Histopathological features were then examined under an Olympus CX-31 microscope (Evident Corp., Tokyo, Japan) (23).

#### 2.10.4 Mouse complete blood count

Blood from the sinus was placed into EP tubes with anticoagulant. Then the results were seen through automatic hematology machinery for the quantity of red blood cells (RBC), white blood cells (WBC), lymphocytes (LYM), and platelets (PLT) (24).

#### 2.10.5 Determination of cytokine secretion levels and immunoglobulins in serum

Bleeding was carried out from retro-orbital sinus of the mice through capillary glass tube in Eppendorf (EP) microcentrifuge tube. And then they were spun at 10,000 rpm for 10 min and the serum was softly split from the clot. Serum interleukin-6 (IL-6), interleukin-2 (IL-2), interferon-*γ* (IFN-γ), tumor necrosis factor-*α* (TNF-α), immunoglobulin E (IgE), and immunoglobulin G (IgG) were measured using mouse ELISA kits (BioLegend, ELISA MAX™ Deluxe Set; Cat# 431004, 431,304, 430,804, 430,904, 432,404, and 479,604) according to the manufacturer’s instructions. Samples were assayed in triplicate. The absorbance of blank wells was subtracted prior to standard curve fitting, and concentrations were calculated from the standard curve (24).

#### 2.10.6 Preparation of splenocyte suspensions

The spleens were removed from the euthanized animals. Then each spleen was rinsed gently in cold PBS to get rid of any remaining blood, and was trimmed of fat and connective tissue. The tissue was then gently pressed through a 200-mesh stainless-steel sieve using a sterile syringe plunger to obtain a cell suspension. The suspension was washed with PBS and placed onto a mouse lymphocyte separation medium. After centrifugation at 2,000 rpm for 15 min at 4 °C, the lymphocyte layer was collected and washed twice with PBS. Finally, the cells were resuspended in RPMI-1640 medium and adjusted to a single-cell suspension for further analyses (25).

#### 2.10.7 Determination of spleen lymphocyte phenotype

For T-cell phenotyping, 100 μL of the splenocyte suspension was added to flow cytometry tubes. The cells were incubated with fluorochrome-labeled anti-CD3, anti-CD4, and anti-CD8 antibodies for 30 min at room temperature in the dark. And resuspended to a final concentration of 1.0 × 10^5^ cells/mL. Flow cytometry was performed within 1 h. Populations of CD3^+^, CD3^+^CD4^+^, and CD3^+^CD8^+^ T cells were measured, and the CD4^+^/CD8^+^ ratio was calculated (25, 26).

#### 2.10.8 Apoptosis in mouse spleen cells

Apoptosis was measured using Annexin V-FITC and propidium iodide (PI) staining. Cells were washed twice with cold PBS and resuspended in 1 × binding buffer at a density of 1.0 × 10^5^ cells/mL. For each sample, 100 μL of the suspension was incubated with 5 μL Annexin V-FITC and 5–10 μL PI for 15 min at room temperature in the dark. Stained for 400 μL of 1 × binding buffer. Then we used flow cytometry to detect the samples after an hour. Early and late apoptotic cells were separated based on Annexin V and PI fluorescence signals (27).

### 2.11 Statistical analysis

Experiment results are shown as mean ± s.d. Statistical comparison was done using least significant difference test for the differences. Difference is statistically significant if *p* < 0.05. All data analysis was done by SPSS statistics 26. 0 (IBM SPSS statistics, Armonk, New York, USA) and GraphPad prism 6. 0 (GraphPad software, La Jolla, California, USA).

## 3 Results and discussion

### 3.1 Optimal synthesis conditions

To look for the best ways to make polysaccharide-selenium complexes, a single-factor test was carried out. The result showed that the chelated complex has the most selenium content which is 267.61 μg/g when the reaction time is 4 h, the water bath temperature is 60 °C, and the mass ratio of polysaccharides to sodium selenite is 4:6. Statistical analysis of the data revealed significant differences (*p* < 0.05) between the experimental groups, with the optimal conditions yielding a selenium content that was significantly higher than those obtained under other conditions (Supplementary Figure 4).

### 3.2 Characterization analysis

**Figure 1:**
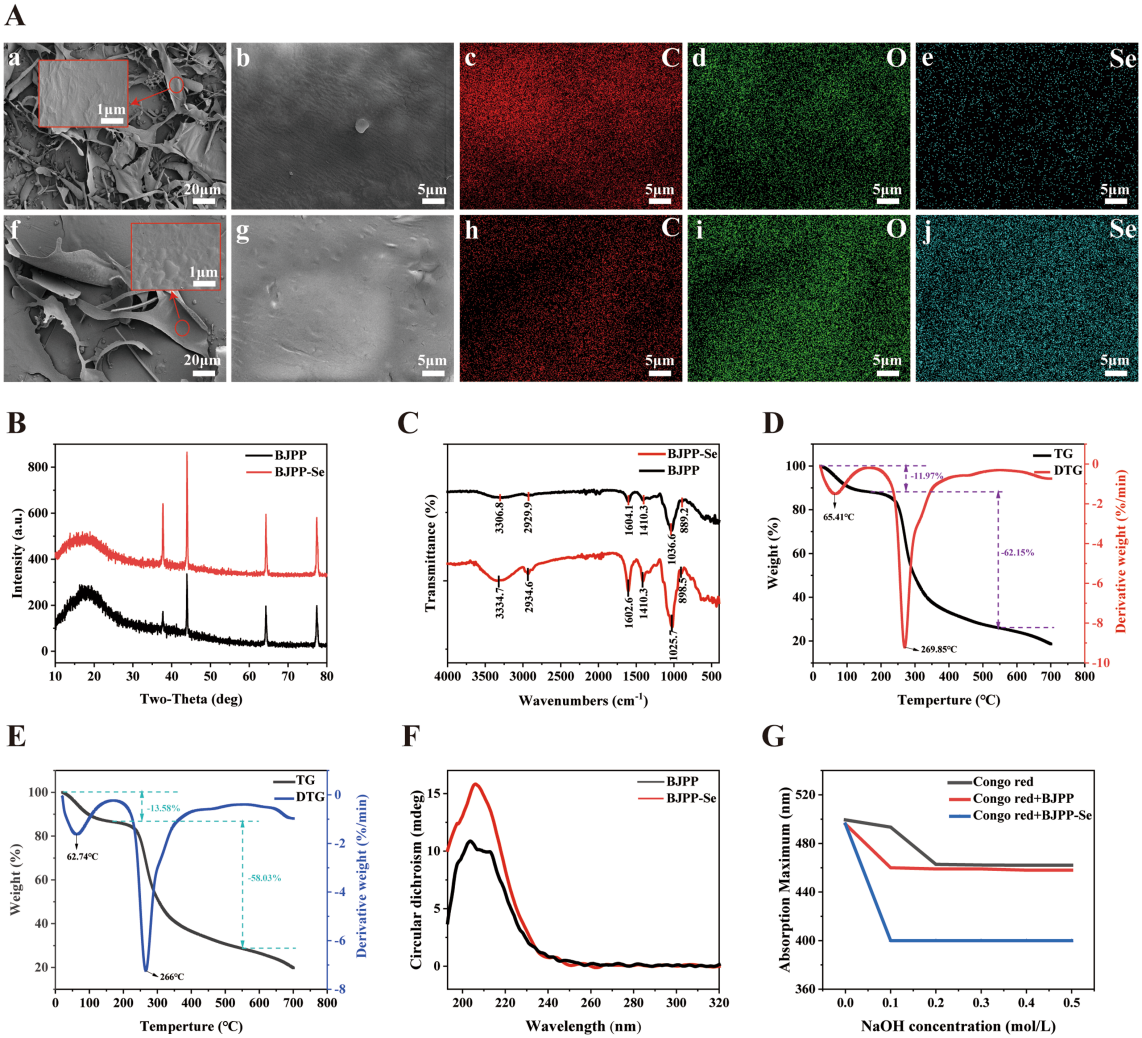
**(A)** SEM-EDS images of BJPP (a–e) and BJPP-Se (f–j); **(B)** XRD; **(C)** FTIR; **(D)** TG and DTG curves of BJPP; **(E)** TG and DTG curves of BJPP-Se; **(F)** CD; **(G)** Congo red experiments.

As can be seen from Figure 1A and Supplementary Figure 5, both BJPP and BJPP-Se were dominated by lamellar structures with some streaks. Compared with BJPP, BJPP-Se became smoother, indicating a change in the structure of the complexed polysaccharides, which may be attributed to an increase molar volume of the selenium-complexed polysaccharides, leading to an increase in the particle size, which in turn caused a change in the surface structure. Further SEM-EDS analysis showed that selenium was absent in BJPP but present in BJPP-Se. Based on EDS semi-quantitative analysis, the surface elemental proportion (atomic %) of Se in BJPP-Se was 19.45%, supporting the successful incorporation of selenium.

XRD analysis can examine whether selenium chelation alters the crystal structure of BJPP. A series of broad “bread-like” peaks are observed in the low-angle region between 10° and 25° (2θ). These diffuse peaks usually indicate amorphous or poorly ordered regions, often attributed to the semi-crystalline nature of some biopolymer systems (28). In contrast, the diffraction pattern also shows four distinct narrow peaks at 37.6°, 44°, 64.5° and 77.7°. From Figure 1B, it can be seen that the crystal structure of BJPP-Se did not change as compared with BJPP but the crystallinity has increased and this is due to the modification of polysaccharides which results in changes in the crystallinity (14).

The native polysaccharide showed typical UV absorption between 190 and 220 nm due to n → σ* electronic transitions of C-O bonds in the saccharide rings and absorption by the terminal hemiacetal oxygen (29). UV–vis spectrum (Supplementary Figure 6) did not show significant absorption between 260 and 280 nm indicating that there is no presence or very less quantity of proteins and nucleic acids in the obtained polysaccharide. On the other hand, BJPP-Se exhibited an obvious red shift of the maximum absorption (from 196 nm to 216 nm). This shift can be rationalized by changes in electronic transitions associated with selenylation: the formation of C-O…Se/selenite-related linkages and the increased polarizability/electron-withdrawing character of selenium alter the local electronic environment of the glycosidic framework (30), which stabilizes the excited state and reduces the transition energy gap (ΔE), resulting in absorption at longer wavelengths.

FT-IR analysis (Figure 1C) showed several characteristic absorption bands, which confirmed the polysaccharide nature. The O-H stretching vibration peaks of polysaccharides are at 3306.8 cm^−1^ and 3334.7 cm^−1^. The shift in the peak indicates that there may be hydrogen bonding interactions between BJPP and selenium (31, 32). While those at 2929.9 cm^−1^ and 2934.6 cm^−1^ were C-H stretching vibration peaks (33, 34), which were typical characteristic absorption peaks of polysaccharides. The absorption peaks at 1604.1 cm^−1^ and 1602.6 cm^−1^ are C=O stretching vibrations, and these bands may be attributed to conjugated carbonyl groups or to adsorbed water molecules interacting with carbonyl functional groups (35) 1410.3 cm^−1^ is associated with the C-H bending vibration, which is consistent with the backbone vibration in the glycan backbone (33). In addition, the C-O-H absorption peak in the BJPP-Se spectrum appears at 1025.7 cm^−1^, which is lower than the peak at 1036.6 cm^−1^ in BJPP. Based on previous studies (36), it can be inferred that the C-O-H bond in BJPP is broken and combines with selenium to form a new C-O…Se bond (31, 32). And also it can be seen that there are peaks on 889.2, 898.5 cm^−1^ that denote the existence of *β* - glycosidic bonds, which are the main structural parts to decide the 3D configuration and biological performance of polysaccharides (34).

### 3.3 TG analysis

From Figures 1D,E it can be seen that the TG and thermogravimetric derivative (DTG) curves of BJPP and BJPP-Se are the same. In the temperature range of 25–150 °C, significant weight loss was seen for BJPP and BJPP-Se, mainly because of the evaporation of free and bound water of BJPP and BJPP-Se, the weight loss rate is 11.97 and 13.58%, respectively, (37). From the above figure, it is clear that the weight loss rate of BJPP-Se increased rapidly along with the increasing temperature, and the highest weight loss range was 200–550 °C, and the weight loss rate was around 58.03%. Therefore, the decomposition temperature of BJPP-Se is 266 °C, which means that the BJPP-Se rapidly decomposes. Similarly, BJPP decomposes violently in the range of 200–550 °C with a peak value of 269.85 °C and the mass loss rate of BJPP is about 62.15% (29). From the above analysis, it can be concluded that the thermal stability of BJPP-Se is basically unchanged compared to BJPP. In the final step, the two samples were carbonized, yielding 25.88 and 28.39% residual weights of BJPP and BJPP-Se, respectively. Similar TG and DTG of BJPP and BJPP-Se shows that Se incorporation does not obviously change their thermal degradation behaviors.

### 3.4 Structural analysis

In this study, CD spectroscopy was carried out on the electromagnetic spectrum in the UV wavelength region (190–330 nm) to detect changes in conformation of polysaccharides before and after selenylation (38), as shown in Figure 1F. There was a positive cotton effect at 203 nm and 205 nm, so it can be concluded that BJPP and BJPP-Se are ordered. This is in agreement with the previous reports about selenium-enriched polysaccharides (14).

Congo Red is a well-established dye that forms complexes with biomolecules having a triple-helix conformation, resulting in a shift in the maximum absorption wavelength (39). Enables further observation of the helical structure of polysaccharides. This spectral variation suggests that neither BJPP nor BJPP-Se has a triple-helical conformation under the conditions tested (28). This is consistent with the results of CD, demonstrating the absence of helical structures in BJPP and BJPP-Se (Figure 1G).

The number average molecular weights of BJPP and BJPP-Se were determined to be 88.263 kDa and 74.322 kDa, respectively (Supplementary Table 1). This reduction is most likely attributable to acidolysis (acid-catalyzed cleavage/hydrolysis of glycosidic bonds) under the acidic HNO_3_-Na_2_SeO_3_ selenylation conditions, (12), which leads to chain shortening and a decreased average Mw (40). This explanation is consistent with our synthesis conditions (0.6% HNO₃ with thermal input during selenylation). The decrease in molecular weight may contribute, at least in part, to the enhanced bioactivity of BJPP-Se. In general, moderate chain-length shortening can improve aqueous dispersion/diffusion and increase the accessibility of interactive sites, which may facilitate interactions with immune-relevant cell-surface components and downstream signaling. Nevertheless, we emphasize that the improved activity is unlikely driven by Mw reduction alone; rather, it likely reflects the combined effects of chain-length shortening together with selenium-induced overall structural reorganization. BJPP and BJPP-Se are mainly composed of Fuc, Rha, Ara, Gal, Glc, Xyl, Gal-UA, and Glc-UA. The molar mass ratios of BJPP were 1.24:33.36:23.61:18.91:2.20:5.45:13.95:1.29. The molar mass ratio of BJPP-Se was 1.21:35.69:24.16:19.68:2.16:5.19:10.85:1.06 (Supplementary Figure 7 and Supplementary Table 2). The similarity of the monosaccharide compositions indicated that the modification process did not change the main composition of the polysaccharides (24), while only minor variations in molar ratios were observed, which are likely attributable to acid-related structural effects during selenylation and routine variability associated with hydrolysis and measurement.

### 3.5 XPS analysis

**Figure 2:**
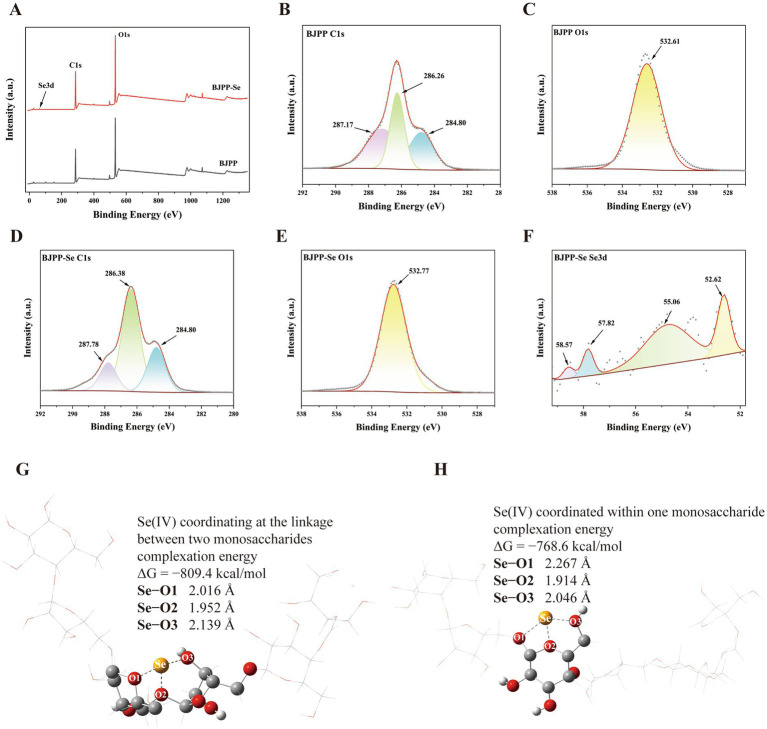
XPS patterns of BJPP and BJPP-Se: **(A)** XPS spectrum of BJPP and BJPP-Se; **(B)** high-resolution C 1 s spectrum of BJPP; **(C)** high-resolution O 1 s spectrum of BJPP; **(D)** high-resolution C 1s spectrum of BJPP-Se; **(E)** high-resolution O 1 s spectrum of BJPP-Se; **(F)** high-resolution Se 3d spectrum of BJPP-Se. Two coordination configurations of Se (IV) in monosaccharide systems. **(G)** Coordinating at the linkage between two monosaccharides; **(H)** coordinated within one monosaccharide.

XPS was further used to confirm the surface presence and chemical states of selenium in BJPP-Se. As shown in Figure 2A, there is a Se 3d peak in the XPS scanning of BJPP-Se, which proves that selenium is successfully incorporated into the BJPP structure. However, since the content of carbon and oxygen in the sample is very rich, the detection depth is great, so the Se 3d signal is relatively weak. The deconvoluted Se 3d spectrum can be seen in Figure 2F. The Se^0^ element peak appears at 55.06 eV, and the Se^4+^ element peak is found at 58.57 eV (41). This indicates a valence transformation of selenium from +4 to 0, resulting in the coexistence of Se^0^ and Se^4+^ species. The findings suggest that, after selenization, selenium predominantly exists in its elemental state, while a minor fraction remains as selenite residues bound to the polysaccharide chains. The presence of Se^0^ accounts for the amorphous character observed in the XRD pattern (41). Compared with Se^4+^, Se^0^ exhibits lower toxicity and superior antioxidant capacity (42). In the C 1 s spectra from both BJPP and BJPP-Se in Figures 2B,D more than two different carbon states can be seen. In terms of data for the BJPP, the peaks at 284.80 eV, 286.26 eV and 287.17 eV represent, respectively, the C=O, C-OH bond and bond C-H from the structure (32). Moreover, comparing the O 1 s spectra of BJPP and BJPP-Se (Figures 2C,E) shows a small shift in the binding energy, from 532.61 eV for BJPP to 532.77 eV for BJPP-Se. This suggests possible C-O…Se bonds are forming. The C-O…Se bonds help to reduce the electron density around the oxygen atoms, this reduces the shielding of the electrons causing an increase in the binding energy (16). They correspond to those from the FT-IR spectra. Thus, from the XPS it gives information on the chemistry around selenium in BJPP-Se and therefore what exactly selenium is doing in the BJPP matrix.

### 3.6 The coordination pattern of Se (IV) with BJPP

Besides experimental observations, DFT calculations offered additional confirmation of the interaction between BJPP and selenium ions. Two representative coordination configurations were observed (Figures 2G,H): one where Se (IV) bridges two monosaccharide units, and another limited to a single monosaccharide. Inter-monosaccharide coordination had a lower Gibbs free energy (−809.4 kcal/mol) compared to the intra-molecular mode (−768.6 kcal/mol), which implies it is more stable. This result implies that Se (IV) prefers to adopt a bridging coordination mode, thus improving the electronic coupling between two neighboring sugar residues.

Specifically, the DFT-predicted preference for the bridging motif is consistent with the experimentally observed changes in BJPP-Se relative to the native polysaccharide (e.g., selenium incorporation signals and accompanying structural features revealed by our characterization), together with the enhanced immunomodulatory performance. In this view, the motif where Se (IV) bridges two monosaccharide units more likely to couple neighboring residues and thereby contribute to broader structural reorganization (e.g., conformational/aggregation changes) that could affect bio interface presentation, whereas the motif limited to a single monosaccharide may mainly influence local microenvironments around selenium-associated sites.

### 3.7 Effects of BJPP and BJPP-Se on CTX-modeling immunodeficient mice

#### 3.7.1 Changes in body weight and effects of BJPP and BJPP-Se on spleen and thymus indices

**Figure 3:**
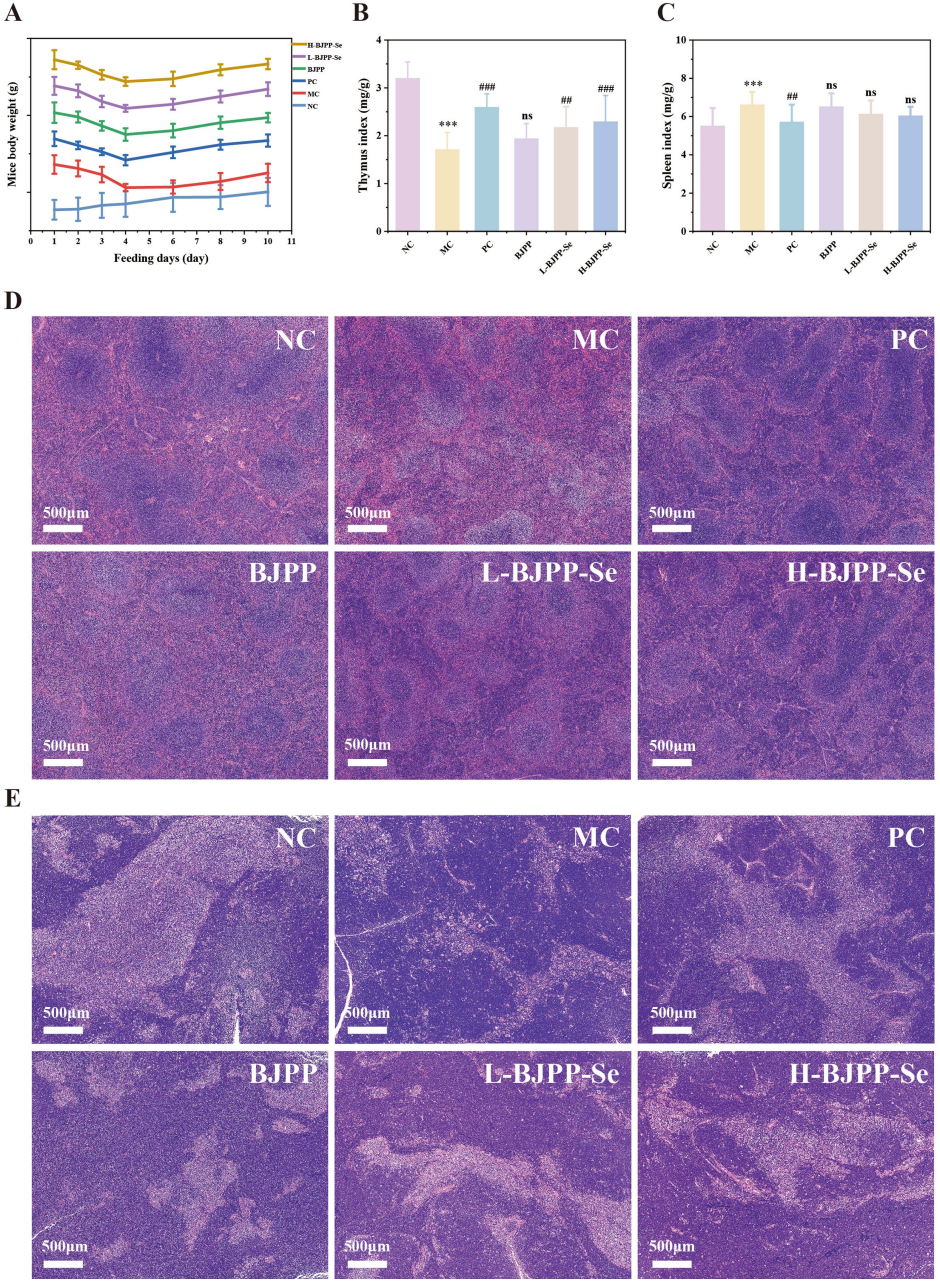
**(A)** Mouse body weight curve during the experiment; **(B)** thymus index; **(C)** spleen index. Values are presented as mean ± SD (*n* = 12). **(D)** HE staining of spleen; **(E)** HE staining of thymus (scale bar = 500 μm). Statistical analysis was performed using one-way ANOVA followed by the least significant difference (LSD) *post hoc* test. ^*^p < 0.05, ^**^p < 0.01, ^***^p < 0.001 vs. NC; ^#^*p* < 0.05, ^##^*p* < 0.01, ^###^*p* < 0.001 vs. MC; ns, not significant.

Figure 3A is the weight changes for the mice over the course of the experiment. Within 3 days post-intraperitoneal CTX injection, there were notable signs of weight loss as well as poor hair and laziness compared to the control group of mice which had not been treated and thus demonstrates successful creation of the model of immunosuppression. By day 10, all polysaccharide - treated groups had significantly increased body weight (bw) compared to the model group, and the effect was more significant in the BJPP-Se and BJPP groups.

Immune organs are indicators of a host’s immune status, and their indices are an immediate measurement of change (43). From Figure 3B, the thymus index of the MC group is significantly lower than that of the NC group, indicating that the immunosuppression induced by CTX has been successfully constructed. Levamisole Hydrochloride brings a clear recovery of thymus index (44), which can reverse CTX induced thymic damage. BJPP and BJPP-Se treatments also increased the thymus index, and the improvement became more pronounced with higher doses, indicating a dose-dependent protective effect. Figure 3C shows that the spleen index in the MC group was significantly higher than that in the NC group. CTX often causes inflammatory swelling and structural disruption of the spleen, and this pattern is consistent with previous reports (26). All treatment groups showed some suppression of spleen enlargement. However, only PC group and H-BJPP-Se group achieved significant reductions. These results suggest that polysaccharides, particularly when combined with selenium, can mitigate CTX-induced immune organ damage and help restore the functional status of both the thymus and spleen (22).

#### 3.7.2 Effects of the BJPP and BJPP-Se on the morphology of the spleen and thymus

Figure 3D shows the effects of BJPP and BJPP-Se on the morphology of mouse spleens. In the blank control group, the spleen showed clear splenic trabeculae and well-defined disappeared, and the boundaries between RP and WP became unclear, indicating severe spleen injury and confirming successful immunosuppression. Treatment with BJPP reduced these structural abnormalities. The peripheral zone became more defined, and the central arteries and trabeculae were easier to observe. At the same dose, BJPP-Se produced stronger improvements than BJPP. RP and WP were more clearly separated, and the splenic trabeculae were more distinct. Increasing the BJPP-Se dose led to gradual enhancement in WP staining and expansion, suggesting a dose-dependent repair effect.

In terms of the thymus of Figure 3E, it is clear that in the model group, there is an obvious demarcation between the cortex and medulla, but the medullary region has a clear reduction compared to CTX-induced histopathological injury. BJPP and BJPP-Se both decreased these changes as seen by more apparent re-emergence of cortex-medulla boundaries and partial medullary area recovery. Especially at the same dose, BJPP-Se had better protective and regenerative effects on thymic morphology than BJPP.

#### 3.7.3 Effects of BJPP and BJPP-Se on mouse hematopoietic function

**Figure 4:**
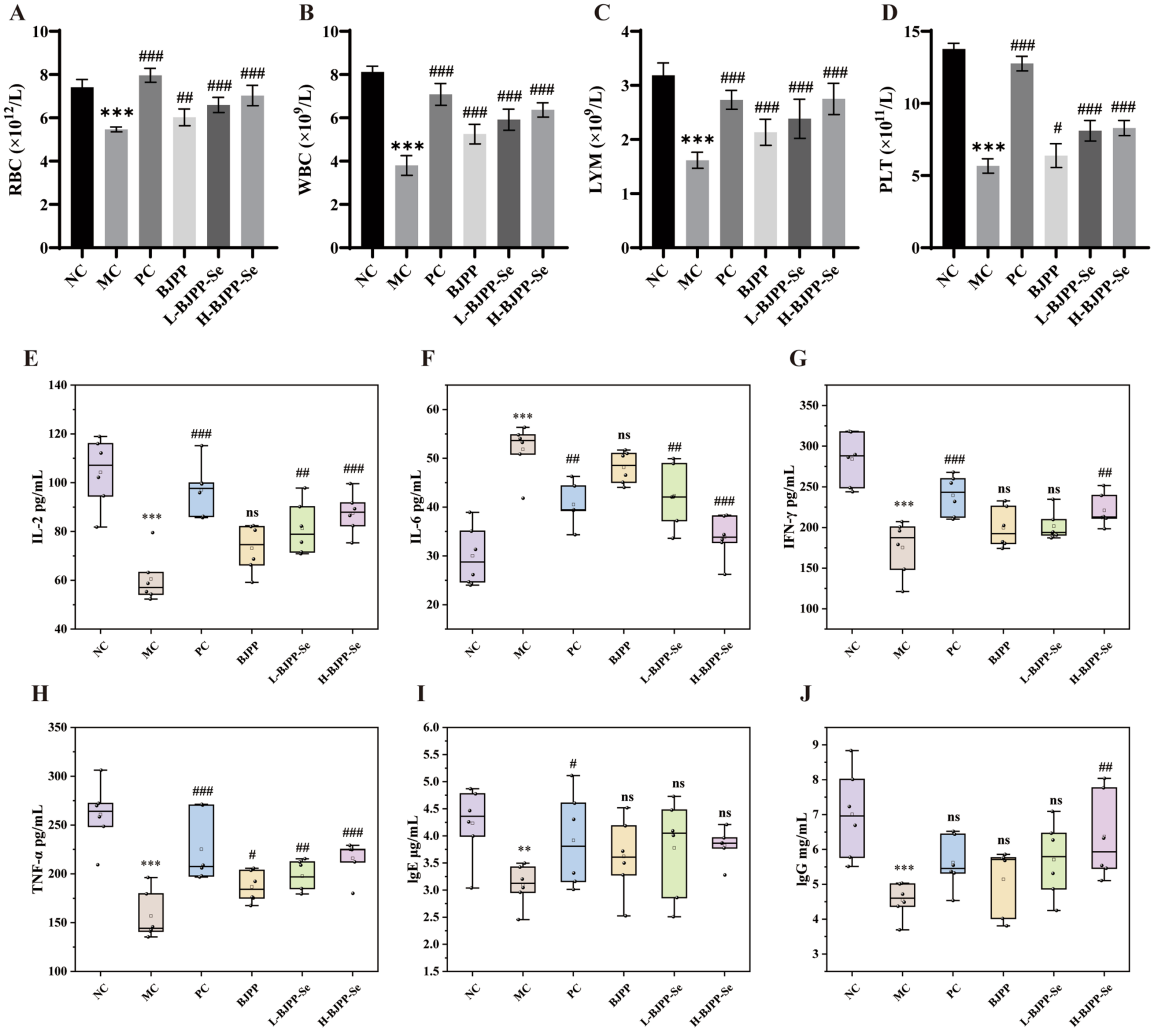
Effects of BJPP and BJPP-Se on mouse hematopoietic function: **(A)** RBC; **(B)** WBC; **(C)** LYM; **(D)** PLT. Effects of BJPP and BJPP-Se on cytokine and immunoglobulin levels: **(E)** IL-2; **(F)** IL-6; **(G)** IFN-γ; **(H)** TNF-α; **(I)** IgE; **(J)** IgG. Values are presented as mean ± SD (*n* = 6). Statistical analysis was performed using one-way ANOVA followed by the least significant difference (LSD) post hoc test. ^*^p < 0.05, ^**^p < 0.01, ^***^p < 0.001 vs. NC; ^#^*p* < 0.05, ^##^*p* < 0.01, ^###^*p* < 0.001 vs. MC; ns, not significant.

CTX is a well-known myelosuppressive drug that reduces peripheral blood cell counts in mice (45). As shown in Figures 4A–D, compared with the NC group, the MC group exhibited significantly reduced levels of RBC, WBC, LYM, and PLT, indicating pronounced bone marrow suppression and systemic immunosuppression. Treatment with BJPP increased all four blood parameters relative to the MC group. These improvements suggest that BJPP can ease CTX-induced myelosuppression and support recovery of hematopoietic and immune function. Similar effects have been reported for other natural polysaccharides that raise RBC, WBC, LYM, and PLT levels in CTX-treated mice (22). BJPP-Se produced greater improvements than BJPP at the same dose. The BJPP-Se groups consistently showed higher RBC, WBC, LYM, and PLT values than the corresponding BJPP groups, although still slightly lower than those in the PC group. The enhancement caused by BJPP-Se also showed a dose-dependent trend, and the high-dose group approached the positive control. This finding is consistent with studies showing that selenium-enriched peptides or selenium–polysaccharide complexes can markedly improve blood parameters and immune function in CTX-induced immunosuppression (46).

#### 3.7.4 Effects of BJPP and BJPP-Se on cytokine and immunoglobulin levels

Cytokines are major actors for immune system (47). Based on their main functions, cytokines are often classified into T helper 1 (Th1) and T helper 2 (Th2) types. Cytokines like IL-2 and IFN-*γ* and TNF-*α* which are Th1-related mostly work to boost up cell mediated immunity. The Th2-related cytokines like IL-4, IL-6 have more association with humoral immunity and antibody generation. There should be a proper equilibrium between Th1 and Th2 responses in order to maintain immune homeostasis. Therefore, we adopt the following Th1/Th2 index as an immunbalance index to study the immunomodulatory effect of BJPP and BJPP-Se (48).


$$\mathrm{Th}1/\mathrm{Th}2=\frac{\mathrm{IL}-2+\mathrm{IFN}-\gamma +\mathrm{TNF}-\alpha }{\mathrm{IL}-6}$$

Figures 4E–H show the effects of BJPP and BJPP-Se on IL-2, IL-6, IFN-γ and TNF-α in CTX-treated mice. The MC group had way less IL-2, IFN-γ and TNF-α levels than the NC group, which shows that CTX stopped the production of Th1-type cytokines and made cell-mediated immunity weaker (49). BJPP-Se induced larger increases in these Th1 cytokines compared with BJPP at the same dose and also showed a clear dose-dependent trend. IL-6 showed an opposite pattern. Its level was significantly higher in the MC group than in the NC group, even though the mice were immunosuppressed. This increase was likely related to CTX-induced tissue injury and the pro-inflammatory nature of IL-6 (50). CTX damages rapidly dividing cells and triggers the release of damage-associated molecular signals (51). These signals activate innate immune cells and stimulate NF-κB-mediated IL-6 production (52). Therefore, the elevated IL-6 in this model may reflect a compensatory inflammatory response to tissue damage rather than improved immunity. After treatment, IL-6 levels fell in all drug groups. The 200 mg/kg BJPP-Se group brought IL-6 close to the NC level. Taken together, the ability of BJPP—and especially BJPP-Se—to raise Th1 cytokines while lowering IL-6 suggests that these preparations help restore Th1/Th2 balance and reduce the CTX-induced inflammatory state. This conclusion can also be drawn from Table 2.

**Table 2:** Effects of BJPP and BJPP-Se treatment on Th1/Th2 in mice.

Treatment	NC	MC	PC	BJPP	L-BJPP-Se	H-BJPP-Se
Th1/Th2	22.27 ± 3.97	7.59 ± 0.72^***^	13.97 ± 1.67^###^	9.57 ± 0.84	11.60 ± 1.95^##^	15.66 ± 1.68^###^

The values are presented as mean ± SD, n-6. Significant differences with the NC group: ***p < 0.001; Significant differences with the MC group: ^##^*p* < 0.01 and ^###^*p* < 0.001.

Figures 4I,J show the effects of BJPP and BJPP-Se on serum IgE and IgG levels. The MC group displayed significantly lower IgE and IgG levels than the NC group, confirming that CTX inhibited antibody production and successfully established an immunosuppressed state. Similar CTX-induced decreases in serum immunoglobulins have been described in other animal studies (53). Both BJPP and BJPP-Se partially restored IgE and IgG in a dose-dependent manner (54). Notably, 200 mg/kg BJPP-Se increased IgG to a level that exceeded the positive control group. These trends agree with reports that bioactive polysaccharides and selenium-containing complexes can enhance immunoglobulin production and improve humoral immunity in CTX-induced immunosuppression. Taken together, our data indicate that BJPP and especially BJPP-Se modulate both cytokine profiles and antibody responses, thereby contributing to the restoration of immune homeostasis in CTX-treated mice (55).

#### 3.7.5 Spleen T lymphocyte subsets

**Figure 5:**
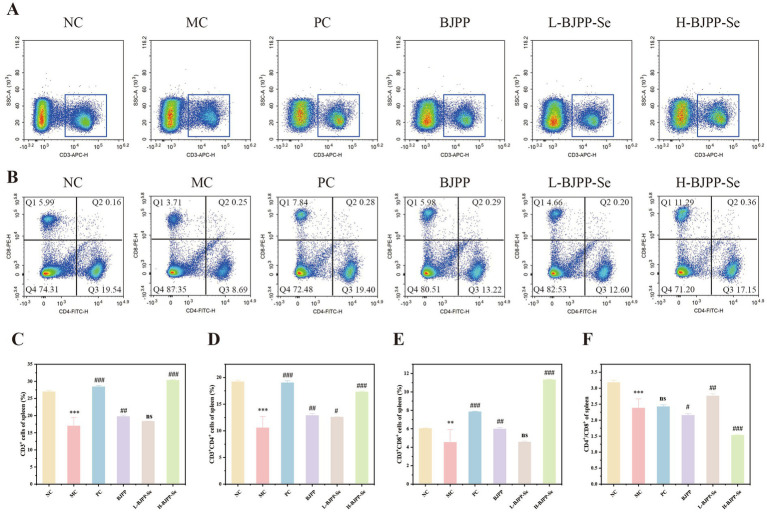
Effects of BJPP and BJPP-Se on mouse spleen cell subpopulations. **(A)** Representative flow cytometry plots of CD3^+^ T cells; **(B)** representative flow cytometry plots of CD4^+^ and CD8^+^ T cells; **(C)** proportion of CD3^+^ cells; **(D)** proportion of CD4^+^ cells; **(E)** proportion of CD8^+^ cells; **(F)** CD4^+^/CD8^+^ ratio. Values are presented as mean ± SD (*n* = 3). Statistical analysis was performed using one-way ANOVA followed by the least significant difference (LSD) post hoc test. ^*^p < 0.05, ^**^p < 0.01, ^***^p < 0.001 vs. NC; ^#^*p* < 0.05, ^##^*p* < 0.01, ^###^*p* < 0.001 vs. MC; ns, not significant.

Lymphocyte function is closely linked to their cell surface markers. CD3 is the main marker for T cells, while mature T cells also express either CD4 or CD8. In adaptive immunity, CD4^+^ T cells act as helper cells and CD8^+^ T cells serve as the main effector cells (26). As shown in Figure 5, the MC group showed clear reductions in CD3^+^, CD4^+^, and CD8^+^ T lymphocyte percentages compared with the NC group, confirming the strong suppressive effect of CTX. Polysaccharide treatment increased these T-cell populations, indicating a recovery of T-cell immunity.

A notable change was observed in the high-dose BJPP-Se group. This group showed a marked rise in CD8^+^ T cells, which lowered the CD4^+^/CD8^+^ ratio. One explanation is the well-known pattern of immune reconstitution after cyclophosphamide injury (56). CTX causes broad lymphocyte depletion, and the body then attempts to rebuild the T-cell pool through homeostatic proliferation (57). During this stage, CD8^+^ T cells usually repopulate more quickly than CD4^+^ T cells. Overall, the strong increase in CD8^+^ T cells in the high-dose BJPP-Se group likely results from a combination of immune reconstitution and immune stimulation, leading to the reduced CD4^+^/CD8^+^ ratio.

#### 3.7.6 Apoptosis in mouse spleen cells

**Figure 6:**
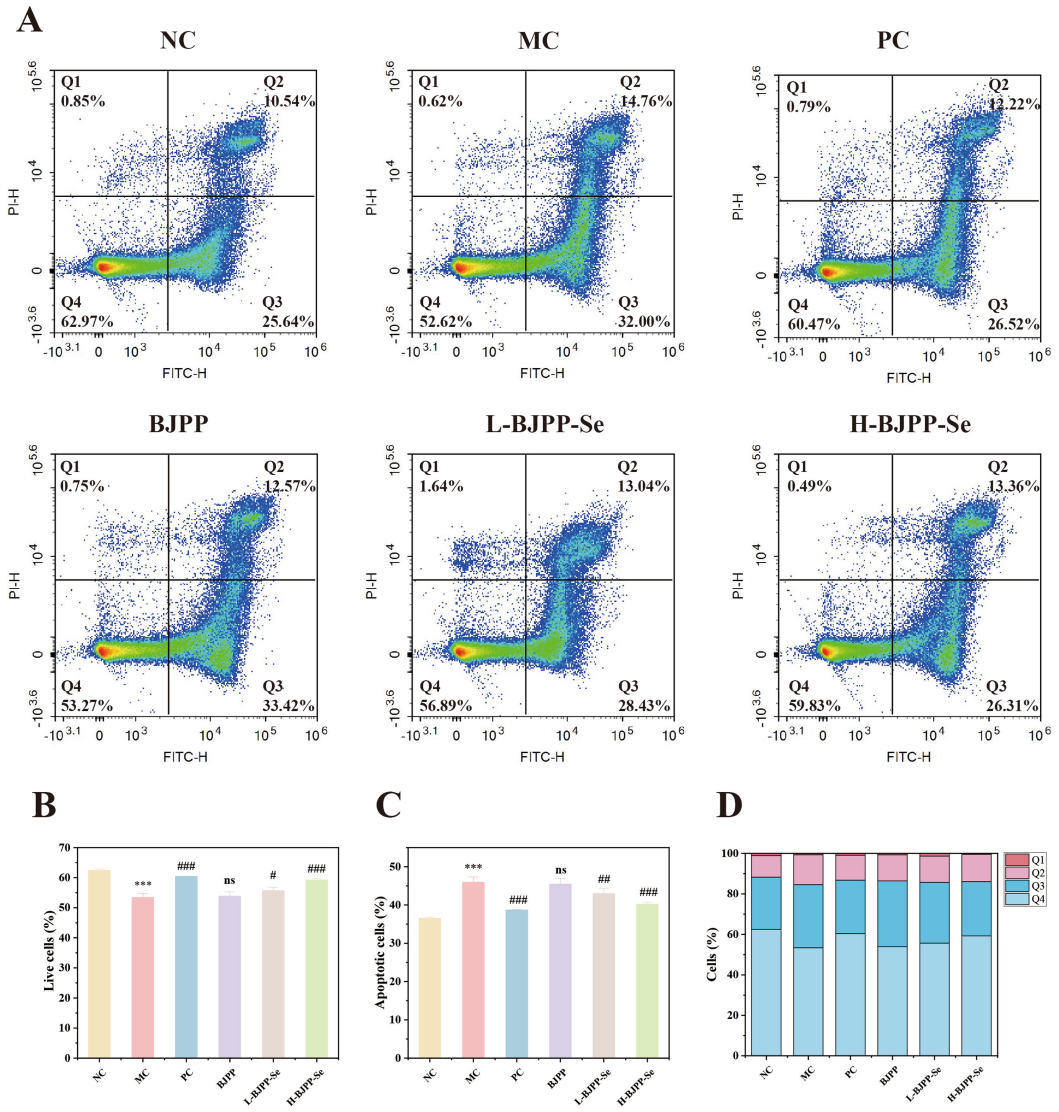
Effects of BJPP and BJPP-Se on apoptosis in mouse spleen cells. **(A)** Representative flow cytometry plots of apoptosis; **(B)** percentage of live cells; **(C)** percentage of apoptotic cells; **(D)** Proportion distribution of the four stages of apoptosis in each treatment group. Values are presented as mean ± SD (*n* = 3). Statistical analysis was performed using one-way ANOVA followed by the least significant difference (LSD) post hoc test. ^*^p < 0.05, ^**^p < 0.01, ^***^p < 0.001 vs. NC; ^#^*p* < 0.05, ^##^*p* < 0.01, ^###^*p* < 0.001 vs. MC; ns, not significant.

The apoptosis data were consistent with the flow cytometry results for T-cell subsets. As shown in Figure 6, in the MC group, the proportion of apoptotic splenic cells was markedly higher than in the NC group. Both early and late apoptotic cells increased, showing that cyclophosphamide caused pronounced injury to immune cells (58).

After treatment, apoptosis rates declined to different extents in all drug groups, suggesting a partial recovery of T-cell populations. The PC group showed a clear reduction in total apoptosis, although the level remained slightly above that of the normal control. Polysaccharides and polysaccharide-selenium groups also decreased the number of apoptotic cells and raised the amount of living cells, bringing it closer to the blank control group. Polysaccharide preparations can protect immune cells from cyclophosphamide - induced apoptosis as shown by all these results (59).

### 3.8 Limitations and future perspectives

This study has two main limitations. First, the current findings are based on a representative batch, and a systematic multi-batch statistical evaluation of reproducibility has not yet been conducted. Second, because the work was performed at laboratory scale, key system boundaries and engineering-scale process data required for reliable techno-economic assessment (TEA) and cost–benefit analysis is not yet available. To address these gaps, future work will prepare multiple independent batches and quantify within- and between-batch variability using a CQA-oriented approach. In parallel, we will progressively establish mass/energy inventories under scale-up conditions, including recovery yields, unit energy demands, and waste handling data, and apply sensitivity analyses to identify key cost drivers, thereby developing a reusable framework for economic evaluation.

## 4 Conclusion

In this study, a selenium-functionalized polysaccharide (BJPP-Se) was synthesized from blackened jujube pomace using an optimized HNO_3_-Na_2_SeO_3_ selenylation process. Selenylation was accompanied by measurable structural changes, supported by selenium-associated linkages (e.g., C-O…Se/selenite-associated bonding) and coordination features indicated by DFT, together with reduced molecular weight and altered supramolecular organization. Functionally, BJPP-Se showed enhanced immunomodulatory performance relative to the native polysaccharide and improved immune-related readouts in an immunosuppressed model without obvious adverse effects under the tested conditions. These observations are consistent with selenium-associated modification of the polysaccharide matrix, which may modulate bioactive motif accessibility and local physicochemical properties; receptor-level validation remains to be established. *In vivo* sites of action and active forms were not determined and warrant future pharmacokinetic and metabolite profiling. The results support biofunctional synergy between the selenium moiety and the polysaccharide matrix and demonstrate a laboratory-scale route for valorizing agro-industrial byproducts into selenium-functionalized polysaccharides. Engineering-scale data are still required for quantitative sustainability/techno-economic evaluation and for rigorous assessment of batch reproducibility and scale-up feasibility.

## Data Availability

The original contributions presented in the study are included in the article/Supplementary material, further inquiries can be directed to the corresponding author.
