# Supplementary material for: Selenium-induced structural reorganization of polysaccharides from blackened jujube pomace enhances immunomodulatory activity
Source: Front Nutr. 2026 Mar 17;13:1791870. doi: 10.3389/fnut.2026.1791870 (PMC13035768; doi:10.3389/fnut.2026.1791870)
Supplement: Supplementary file 1 [file Table_1.docx]

Supplementary Material

# Supplementary Figures and Tables

## Supplementary Figures

## Supplementary Figure 1. Elution profile of blackened date pomace polysaccharide on DEAE-Bestarose FF cellulose ion exchange columns.

**Supplementary Figure 2.** Elution profile of blackened date pomace polysaccharides on Chromdex 75PG dextran gel columns.


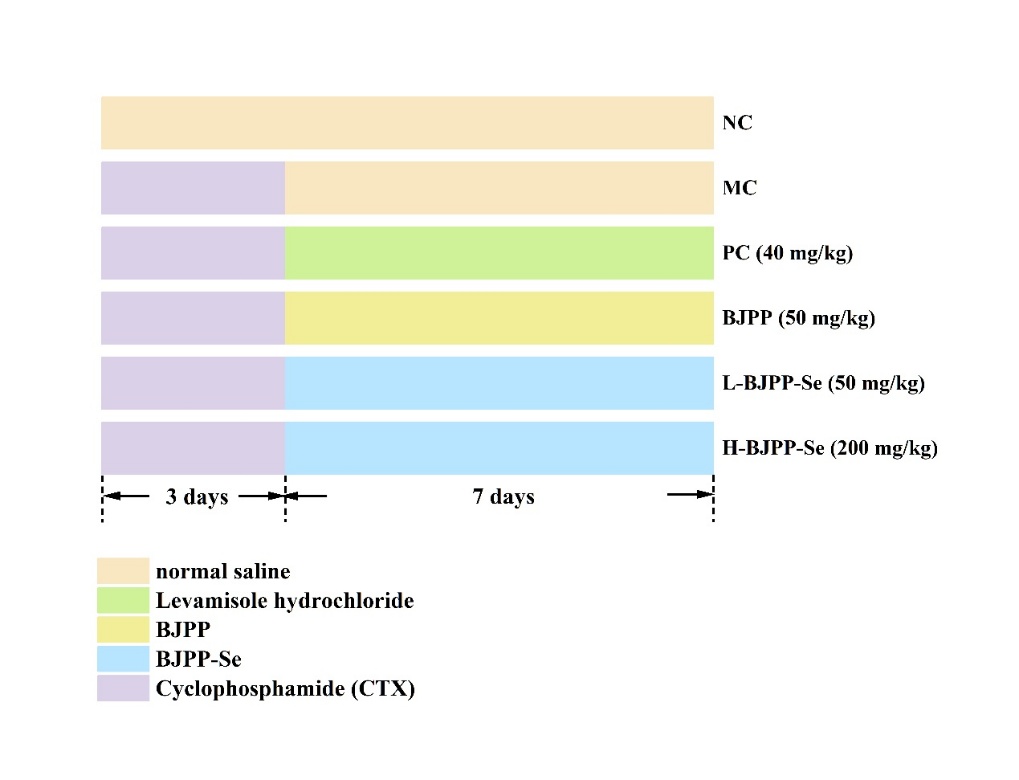


**Supplementary Figure 3.** Animal experiment design scheme.

**
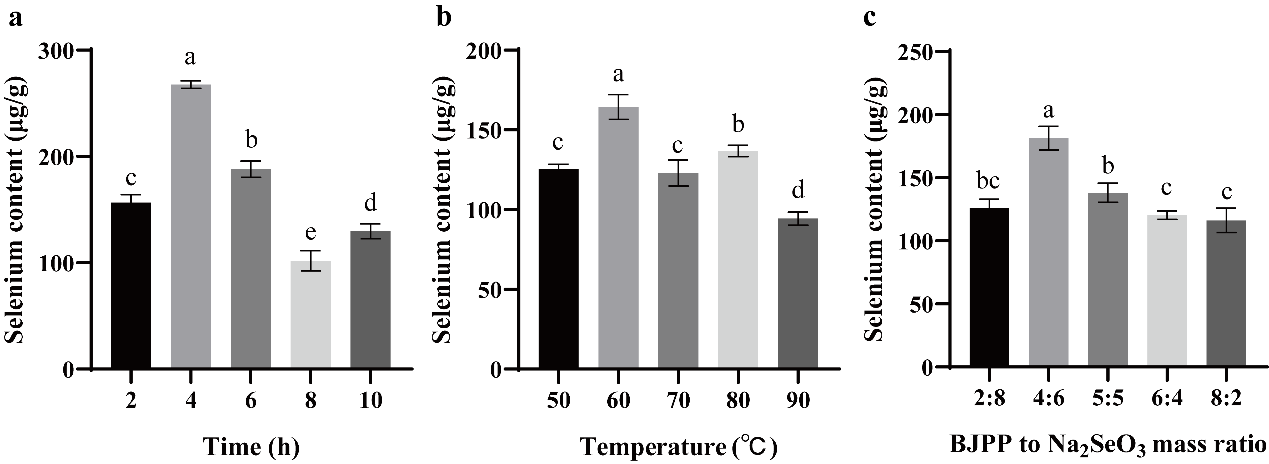
**

**Supplementary Figure 4.** Investigation on the BJPP-Se with different (a) time; (b) temperature; (c) BJPP and Na_2_SeO_3_ mass ratio. Values with different letters denote the significant differences (*p* < 0.05).

**
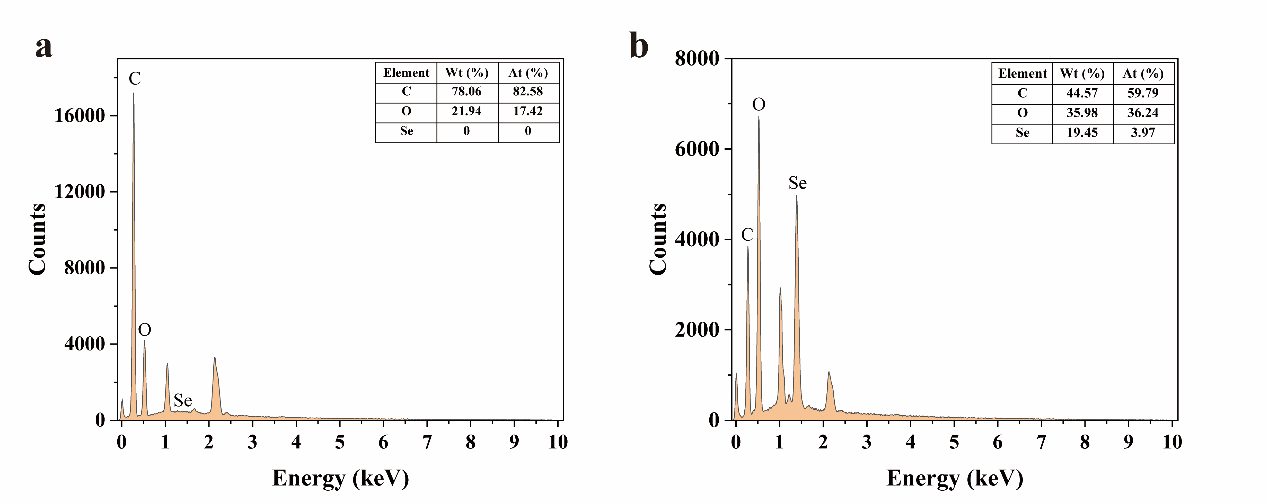
**

**Supplementary Figure 5.** Spectrogram of the total number of distribution maps: (a)BJPP; (b)BJPP-Se.


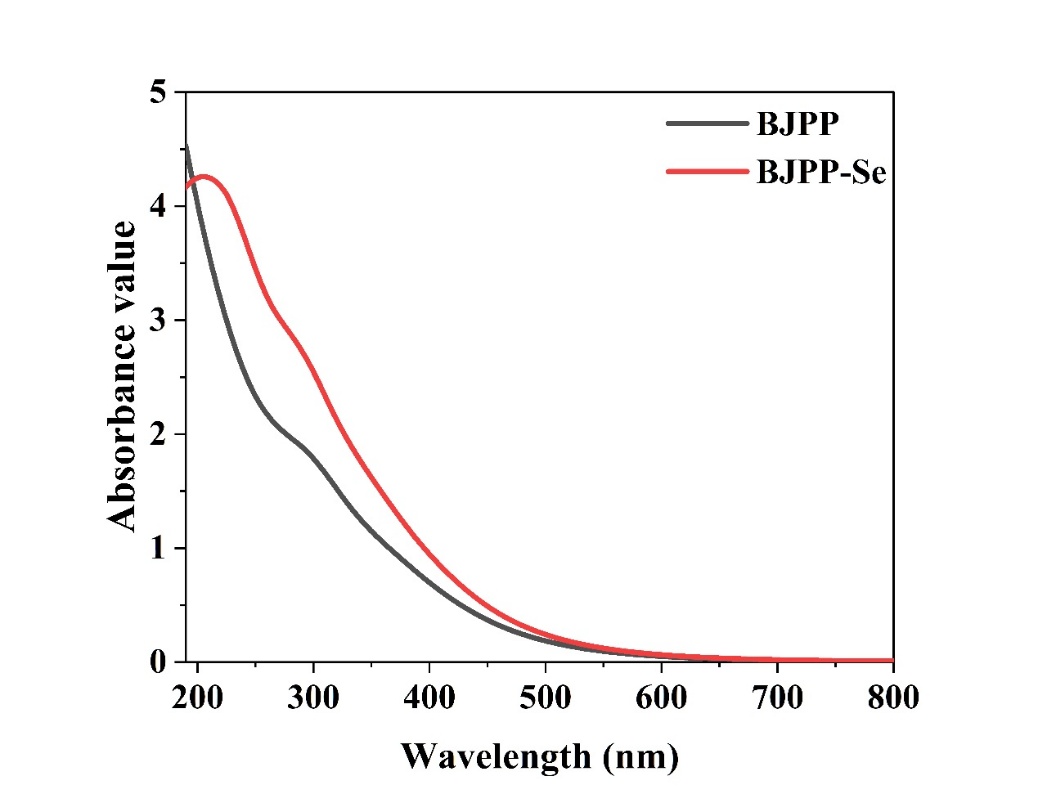


**Supplementary Figure 6.** UV scanning spectra of BJPP and BJPP-Se.


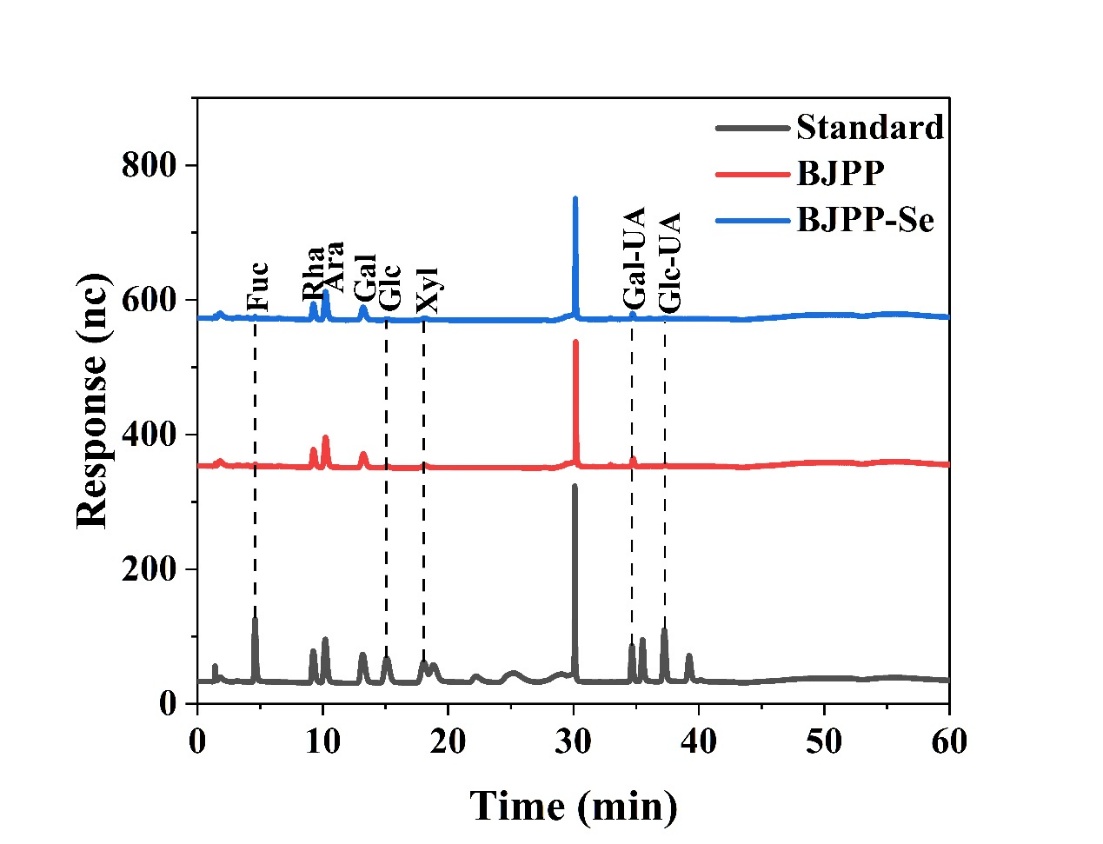


**Supplementary Figure 7.** Monosaccharide Composition of BJPP and BJPP-Se.

## Supplementary Tables

**Supplementary Table 1.** Molecular weights of BJPP and BJPP-Se.

|  | Mn (kDa) | Mw (kDa) | Mz (kDa) | Polydispersity (Mw/Mn) |
| --- | --- | --- | --- | --- |
| BJPP | 88.263 | 125.114 | 193.299 | 1.418 |
| BJPP-Se | 74.322 | 116.821 | 213.195 | 1.572 |

**Supplementary Table 2.** BJPP and BJPP-Se monosaccharide composition.

| Types of monosaccharides | Monosaccharide content | |
| --- | --- | --- |
|  | BJPP (μg/ml) | BJPP-Se (μg/ml) |
| Fuc | 0.6089 | 0.521 |
| Rha | 11.5582 | 10.4159 |
| Ara | 14.9345 | 14.0755 |
| Gal | 10.1581 | 9.3143 |
| Glc | 1.1794 | 1.0215 |
| Xyl | 2.4383 | 2.0452 |
| Man | 0 | 0 |
| Fru | 0 | 0 |
| Rib | 0 | 0 |
| Gal-UA | 8.0781 | 5.5328 |
| Gul-UA | 0 | 0 |
| Glc-UA | 0.7473 | 0.5416 |
| Man-UA | 0 | 0 |
